# Supplementary material for: Multi‐dataset identification of innovative feature genes and molecular mechanisms in keratoconus
Source: J Cell Mol Med. 2024 Sep 19;28(18):e70079. doi: 10.1111/jcmm.70079 (PMC11412914; doi:10.1111/jcmm.70079)
Supplement: Supplementary file 2 — Material S2. [file JCMM-28-e70079-s002.zip › 7.GSEA analysis/3.DBNDD1/KEGG_TERPENOID_BACKBONE_BIOSYNTHESIS.html]

Details for gene set KEGG\_TERPENOID\_BACKBONE\_BIOSYNTHESIS[GSEA]

|  || Dataset | DBNDD1.group.cls#h\_versus\_l |
| Phenotype | group.cls#h\_versus\_l |
| Upregulated in class | h |
| GeneSet | KEGG\_TERPENOID\_BACKBONE\_BIOSYNTHESIS |
| Enrichment Score (ES) | 0.5239995 |
| Normalized Enrichment Score (NES) | 1.3742281 |
| Nominal p-value | 0.13663366 |
| FDR q-value | 0.75114 |
| FWER p-Value | 0.909 |
Table: GSEA Results Summary

  

Fig 1: Enrichment plot: KEGG\_TERPENOID\_BACKBONE\_BIOSYNTHESIS      
 Profile of the Running ES Score & Positions of GeneSet Members on the Rank Ordered List

  

| SYMBOL | TITLE | RANK IN GENE LIST | RANK METRIC SCORE | RUNNING ES | CORE ENRICHMENT || 1 | HMGCS2 | na | 275 | 0.517 | 0.3618 | Yes |
| 2 | IDI2 | na | 3047 | 0.250 | 0.4606 | Yes |
| 3 | MVD | na | 7308 | 0.118 | 0.4221 | Yes |
| 4 | MVK | na | 8538 | 0.094 | 0.4537 | Yes |
| 5 | PDSS1 | na | 10140 | 0.070 | 0.4574 | Yes |
| 6 | IDI1 | na | 10692 | 0.062 | 0.4859 | Yes |
| 7 | PMVK | na | 10962 | 0.059 | 0.5201 | Yes |
| 8 | HMGCR | na | 11996 | 0.047 | 0.5240 | Yes |
| 9 | FDPS | na | 13659 | 0.033 | 0.4996 | No |
| 10 | ACAT1 | na | 14381 | 0.027 | 0.4983 | No |
| 11 | GGPS1 | na | 15116 | 0.022 | 0.4929 | No |
| 12 | ACAT2 | na | 15183 | 0.021 | 0.5064 | No |
| 13 | HMGCS1 | na | 16203 | 0.014 | 0.4874 | No |
| 14 | PDSS2 | na | 19238 | -0.006 | 0.4045 | No |
| 15 | DHDDS | na | 24144 | -0.058 | 0.3049 | No |
Table: GSEA details [plain text format]

  

Fig 2: KEGG\_TERPENOID\_BACKBONE\_BIOSYNTHESIS      
 Blue-Pink O' Gram in the Space of the Analyzed GeneSet

  

Fig 3: KEGG\_TERPENOID\_BACKBONE\_BIOSYNTHESIS: Random ES distribution      
 Gene set null distribution of ES for **KEGG\_TERPENOID\_BACKBONE\_BIOSYNTHESIS**
